# A Comprehensive Review of Advanced Biomarkers for Chronic Kidney Disease in Older Adults: Current Insights and Future Directions

**DOI:** 10.7759/cureus.70413

**Published:** 2024-09-28

**Authors:** Utkarsh Pradeep, Anjalee Chiwhane, Sourya Acharya, Varun Daiya, Paschyanti R Kasat, Pratiksha Sachani, Smruti A Mapari, Gautam N Bedi

**Affiliations:** 1 General Medicine, Jawaharlal Nehru Medical College, Datta Meghe Institute of Higher Education and Research, Wardha, IND; 2 Radiodiagnosis, Jawaharlal Nehru Medical College, Datta Meghe Institute of Higher Education and Research, Wardha, IND; 3 Obstetrics and Gynecology, Jawaharlal Nehru Medical College, Datta Meghe Institute of Higher Education and Research, Wardha, IND

**Keywords:** advanced biomarkers, chronic kidney disease, fibrosis, inflammation, older adults, tubular injury

## Abstract

Chronic kidney disease (CKD) is a growing health concern, particularly in older adults, due to its high prevalence and association with increased morbidity and mortality. Early detection and effective management are crucial to slowing disease progression and reducing complications such as cardiovascular events and end-stage renal disease (ESRD). Traditional biomarkers, including serum creatinine and estimated glomerular filtration rate (eGFR), often have limitations in older populations, where age-related physiological changes can obscure early signs of kidney dysfunction. Advanced biomarkers offer a more precise and comprehensive understanding of kidney health, providing insights into pathological processes such as inflammation, fibrosis, oxidative stress, and tubular injury. These biomarkers have the potential to enhance early diagnosis, predict disease progression, and inform personalized treatment approaches, particularly in the elderly. This review explores the current landscape of advanced biomarkers for CKD in older adults, highlighting their clinical utility and limitations. Key biomarkers, including those related to inflammation (C-reactive protein, interleukin-6), fibrosis (transforming growth factor-beta, collagen degradation products), oxidative stress (F2-isoprostanes, malondialdehyde), and tubular injury (kidney injury molecule-1, neutrophil gelatinase-associated lipocalin), are examined in the context of CKD. Emerging technologies, such as multi-omics and machine learning, are also discussed as they offer new opportunities for biomarker discovery and integration into clinical practice. While challenges remain, including the need for longitudinal studies and better standardization, advanced biomarkers hold promise for transforming CKD management in older adults, paving the way for earlier detection, better risk stratification, and more targeted therapeutic interventions.

## Introduction and background

Chronic kidney disease (CKD) is a significant public health issue, particularly affecting the older population [1]. As the global population ages, the prevalence of CKD has increased significantly, impacting an estimated 10-15% of adults worldwide, with a disproportionately higher burden among older individuals. CKD is characterized by a gradual decline in kidney function, often progressing silently until reaching advanced stages [2]. This is especially problematic in older adults, where the natural, age-related decrease in kidney function can make distinguishing between normal aging processes and pathological kidney damage difficult. Moreover, older adults frequently have comorbid conditions, such as hypertension, diabetes, and cardiovascular diseases, which not only contribute to CKD onset and progression but also worsen outcomes [3]. The burden of CKD in the elderly population is considerable due to its association with increased risks of cardiovascular disease, mortality, and progression to end-stage renal disease (ESRD). The complexity of managing CKD in older adults lies in the need to balance appropriate interventions against the risks of overtreatment, particularly in frail individuals or those with limited life expectancy [4]. In this context, early detection of CKD plays a vital role in managing the disease effectively. Identifying CKD in its early stages allows for implementing preventive measures and therapeutic strategies to slow disease progression, improve the quality of life, and minimize complications in this vulnerable group [5].

Early detection of CKD is crucial to altering its trajectory, as timely interventions can significantly slow the progression of kidney damage and reduce complications such as cardiovascular events and ESRD. In older adults, detecting CKD at an early stage is even more critical, given the subtle and gradual decline in kidney function that occurs with aging [6]. Effective management strategies, including optimizing blood pressure control, managing diabetes, and promoting healthy lifestyle changes, can prevent or delay the progression of CKD when implemented early. However, the challenge lies in accurately diagnosing CKD in older adults, where physiological changes may mask the early signs of kidney dysfunction [7]. Traditional diagnostic markers, such as serum creatinine and estimated glomerular filtration rate (eGFR), have limitations in this population. For instance, serum creatinine levels can be influenced by muscle mass, which tends to decrease with age, potentially leading to an underestimation of kidney impairment. This limitation often results in delayed diagnosis or missed opportunities for early intervention. Consequently, there is a growing need for more reliable and sensitive biomarkers that can accurately detect kidney dysfunction in older adults, particularly in the early stages of CKD when treatment can be most effective [8].

The development and application of advanced biomarkers offer promising avenues for improving CKD diagnosis and management, particularly in older populations. Biomarkers are biological indicators that provide insight into various aspects of kidney health, including function, injury, inflammation, and fibrosis [9]. Unlike traditional markers, which mainly reflect glomerular function, advanced biomarkers provide a broader and more nuanced understanding of kidney health, enabling the detection of early tubular injury, oxidative stress, and other key pathological processes involved in CKD progression [9]. In recent years, numerous biomarkers have emerged as tools for early detection, assessment of disease progression, and prognosis in CKD, particularly for older adults. These biomarkers improve the precision of CKD diagnosis and allow for the stratification of patients based on their risk of progression, enabling more personalized and targeted treatment approaches [10]. Furthermore, biomarkers are valuable in monitoring the efficacy of interventions and guiding clinical decisions, potentially improving outcomes and quality of life for elderly patients with CKD. In this review, we will examine the current state of advanced biomarkers in CKD, their utility in the context of older adults, and the future directions in biomarker research and clinical practice.

## Review

Pathophysiology of chronic kidney disease in older adults

CKD in older adults is a multifaceted condition shaped by age-related physiological changes, common risk factors, and diagnostic complexities. Understanding these factors is essential for effective management and treatment [1]. As people age, the kidneys undergo significant structural and functional changes. One of the most prominent alterations is the decline in glomerular filtration rate (GFR), which decreases by approximately 6-8 ml/min per decade after age 30-40. This reduction is primarily due to a loss of functional nephrons, nephrosclerosis, and diminished renal blood flow [1]. Additionally, aging kidneys exhibit structural changes such as reduced mass, decreased cortical volume, and increased renal cysts and sclerotic glomeruli. These changes lead to a reduced renal reserve and an increased susceptibility to acute kidney injury. Functionally, older adults often face impairments such as a reduced capacity to concentrate urine, eliminate waste products, and maintain fluid balance, all of which are exacerbated by vascular stiffness and altered renal hemodynamics [11]. Several factors contribute to the onset and progression of CKD in older adults, with diabetes mellitus and hypertension being the most common causes. Over time, diabetes can damage renal blood vessels, while hypertension worsens kidney damage by increasing vascular pressure [12]. A history of acute kidney injury (AKI) also significantly elevates the risk of developing CKD later in life. Older adults are especially vulnerable due to pre-existing renal impairment and a reduced functional reserve. Other contributing conditions include glomerulonephritis, polycystic kidney disease, and renovascular disease. Lifestyle factors, such as obesity and smoking, further compound the risk, presenting a multifaceted challenge in this population [13]. Diagnosing CKD in older adults is particularly challenging due to the overlap between normal aging processes and pathological changes associated with CKD [14]. It can be difficult to distinguish between normal age-related kidney function decline and actual kidney damage. Many older adults maintain a normal serum creatinine level despite significant reductions in GFR due to decreased muscle mass. Additionally, CKD often progresses silently, with few symptoms until the disease is in more advanced stages. This makes early detection difficult, as many older adults may not recognize early signs of kidney dysfunction. Current guidelines recommend routine screening for individuals over the age of 60, but many remain undiagnosed until complications develop. Relying on eGFR, without accounting for age-related changes, can also lead to misdiagnosis or delayed intervention [14].

Traditional biomarkers for CKD

The diagnosis and management of CKD traditionally rely on key biomarkers, including serum creatinine, eGFR, blood urea nitrogen (BUN), and the urinary albumin-to-creatinine ratio (UACR). While these biomarkers are essential in assessing kidney function and detecting damage, they have notable limitations, particularly in older adults [15]. Serum creatinine is a common marker used to evaluate kidney function, reflecting a waste product from muscle metabolism. When kidney function declines, serum creatinine levels typically rise. However, factors such as age, gender, and muscle mass can influence these levels, potentially leading to an underestimation of kidney impairment in older adults. Reduced muscle mass, a frequent occurrence in this population, can result in lower serum creatinine levels that do not accurately represent the extent of kidney dysfunction [16]. The eGFR is calculated using serum creatinine levels alongside factors such as age and sex to estimate the kidneys' filtering capacity. While eGFR is a valuable tool, it may not accurately reflect early-stage CKD in elderly patients with low muscle mass or obesity [17]. Alternative eGFR equations using cystatin C have been suggested, but these, too, have limitations. They may fail to capture the nuances of kidney function in older adults, who often present with atypical clinical features [17]. BUN measures the amount of nitrogen in the blood that results from protein metabolism. Elevated BUN levels may indicate impaired kidney function, yet BUN is also influenced by hydration status and dietary protein intake, which reduces its specificity for CKD diagnosis. In older adults, dietary and fluid intake variations can cause fluctuations in BUN levels that may not correlate with actual renal health [18]. The UACR assesses albumin levels in urine relative to creatinine, serving as a sensitive marker for detecting early kidney damage, particularly in diabetic patients. However, UACR can be affected by factors like urinary tract infections or recent exercise, leading to transient elevations that may not reflect chronic kidney damage. This variability complicates the interpretation of UACR in older adults, who may have multiple comorbidities impacting urinary health [19]. Interpreting these traditional biomarkers in older populations presents unique challenges. Age-related changes in muscle mass and body composition can alter serum creatinine and eGFR calculations, often resulting in underestimating CKD severity. Furthermore, older adults frequently have multiple chronic conditions such as diabetes or hypertension that can influence biomarker levels through systemic inflammation or medication effects [20]. The non-specific nature of these biomarkers complicates diagnosis, as they may reflect other physiological changes or conditions common in the elderly rather than kidney disease [20]. Due to these limitations, there is growing emphasis on integrating clinical context and exploring novel biomarkers alongside traditional ones for more accurate CKD assessment in older adults. Research into innovative biomarkers holds promise for improving CKD diagnosis and management in this population, potentially leading to better patient outcomes [21]. Table 1 presents traditional biomarkers for CKD.

**Table 1:** Traditional biomarkers for chronic kidney disease (CKD)

Biomarker	Description	Clinical Relevance	Limitations
Serum Creatinine [22]	A waste product produced from muscle metabolism.	Indicator of kidney function, used to estimate glomerular filtration rate (GFR).	Influenced by muscle mass, age, diet, and other factors, not sensitive for early CKD.
Blood Urea Nitrogen (BUN) [18]	A waste product formed in the liver is excreted by the kidneys.	Measures kidney function and protein metabolism.	It is affected by diet, liver function, and hydration status; it is less specific.
Urinary Albumin/Creatinine Ratio (ACR) [23]	The ratio of albumin to creatinine in urine.	Detects albuminuria, an early marker of kidney damage.	Influenced by conditions like hypertension and diabetes, it requires timed urine collection.
Serum Cystatin C [24]	A protein filtered by kidneys and a marker of GFR.	A more reliable GFR estimate is independent of muscle mass.	Higher cost and limited availability in routine clinical practice.
Serum Electrolytes [25]	Includes sodium, potassium, and bicarbonate levels.	Reflects the kidney's role in electrolyte balance.	It can be influenced by non-renal factors such as medications and diet.
Estimated Glomerular Filtration Rate (eGFR) [26]	Calculated from creatinine or cystatin C levels.	Widely used for staging CKD.	Inaccurate in body size and muscle mass extremes, and the elderly.

Advanced biomarkers for CKD in older adults

CKD presents a significant health challenge, especially in older adults. Identifying advanced biomarkers is critical for early detection, monitoring disease progression, and guiding treatment strategies. Various categories of biomarkers have emerged, each providing unique insights into the underlying pathophysiology of CKD [10]. Inflammatory biomarkers are pivotal in understanding CKD progression. C-reactive protein (CRP) is a well-established marker of systemic inflammation, with elevated levels often signaling a decline in kidney function. Interleukin-6 (IL-6) is another important cytokine linked to inflammation and adverse outcomes in CKD patients. Similarly, tumor necrosis factor-alpha (TNF-α) contributes to kidney damage and disease progression, underscoring the inflammatory nature of CKD [27]. Fibrosis and extracellular matrix remodeling are central to CKD, making biomarkers in this category particularly significant. Transforming growth factor-beta (TGF-β) is a key driver of renal fibrosis, promoting the accumulation of extracellular matrix components that lead to kidney damage. Tissue inhibitors of metalloproteinases (TIMPs) regulate matrix metalloproteinases involved in tissue remodeling and fibrosis within the kidneys. Furthermore, collagen degradation products can signal increased collagen turnover and fibrosis, indicating renal injury [28]. Oxidative stress is another important factor in CKD, with biomarkers such as F2-isoprostanes and malondialdehyde (MDA) reflecting the extent of oxidative damage. F2-isoprostanes indicate lipid peroxidation, while MDA levels signal cellular damage associated with oxidative stress. Both markers are closely tied to CKD severity and offer insights into the mechanisms driving kidney injury [29]. Tubular injury biomarkers are invaluable for assessing renal damage. Kidney injury molecule-1 (KIM-1) is a highly sensitive marker that significantly rises in response to tubular injury. Neutrophil gelatinase-associated lipocalin (NGAL) is another marker that indicates acute tubular injury and holds prognostic value in CKD. Liver-type fatty acid-binding protein (L-FABP) is an additional marker of proximal tubular injury, and its levels correlate with CKD progression [30]. Endothelial dysfunction is frequently observed in CKD, with biomarkers such as asymmetric dimethylarginine (ADMA) and vascular cell adhesion molecule-1 (VCAM-1) shedding light on vascular health. ADMA, an endogenous inhibitor of nitric oxide synthesis, is associated with endothelial dysfunction and increased cardiovascular risk in CKD patients. VCAM-1, which rises with inflammation and endothelial activation, contributes to the cardiovascular complications commonly seen in CKD [31]. Looking forward, novel biomarkers are emerging to deepen our understanding of CKD. Circulating microRNAs have gained attention as potential biomarkers due to their roles in gene regulation and cellular processes related to kidney health. Additionally, advancements in proteomics and metabolomics are opening new avenues for identifying biomarkers that reflect the complex biochemical changes underlying CKD [32]. A summary of advanced biomarkers for chronic kidney disease in older adults is presented in Table 2.

**Table 2:** Advanced biomarkers for chronic kidney disease (CKD) in older adults

Biomarker	Description	Clinical Relevance	Limitations
Neutrophil Gelatinase-Associated Lipocalin (NGAL) [33]	A protein released by damaged kidney tubules.	Early marker of acute kidney injury (AKI) and CKD progression.	Limited availability; influenced by systemic inflammation.
Kidney Injury Molecule-1 (KIM-1) [34]	A type 1 transmembrane protein is expressed in injured proximal tubules.	Indicates tubular injury and CKD severity.	It is not widely used in clinical practice and is affected by non-renal injuries.
Beta-2 Microglobulin (β2M) [35]	A low molecular weight protein filtered by the glomerulus.	Reflects tubular function and glomerular filtration rate (GFR) decline in CKD.	Elevated in inflammation and certain malignancies; not kidney-specific.
Fibroblast Growth Factor-23 (FGF-23) [36]	A hormone involved in phosphate metabolism and vitamin D regulation.	An early marker of mineral bone disorders and cardiovascular risk in CKD.	Elevated in elderly; influenced by dietary phosphate and other factors.
Transforming Growth Factor-Beta (TGF-β) [37]	A cytokine involved in fibrosis and tissue repair processes.	Associated with renal fibrosis and CKD progression.	Systemic marker; not specific to kidney tissue.
Interleukin-18 (IL-18) [38]	A pro-inflammatory cytokine produced by renal tubules during injury.	An early marker of AKI and CKD progression in older adults.	Elevated in systemic inflammation; low specificity.
Procollagen Type III N-terminal Propeptide (PIIINP) [39]	A marker of fibrosis and extracellular matrix turnover.	Indicates renal fibrosis and CKD progression in older adults.	Influenced by systemic fibrotic conditions; limited availability.
Soluble Tumor Necrosis Factor Receptor 1 and 2 (sTNFR1 and sTNFR2) [40]	Soluble forms of TNF receptors are involved in inflammatory pathways.	Predicts CKD progression and mortality risk in older adults.	Elevated in systemic inflammation; limited clinical use.

Biomarkers for predicting CKD progression and outcomes in older adults

Biomarkers are pivotal in predicting the progression and outcomes of CKD in older adults. Identifying and validating these biomarkers can improve risk stratification, prognostication, and monitoring of treatment responses, ultimately enhancing patient care [41]. Traditional biomarkers, such as serum creatinine (SCr) and eGFR, remain standard measures of kidney function. However, these markers have limitations, especially in older adults where reduced muscle mass may distort results. Urinary albumin is another key biomarker; albuminuria is a strong predictor of CKD progression and is frequently used with eGFR for more accurate risk stratification [42]. Beyond traditional markers, several novel biomarkers offer valuable insights into kidney health. KIM-1 is associated with tubular injury and correlates with CKD progression. NGAL is another promising biomarker; elevated levels are linked to declining kidney function and can indicate early damage [9]. Fibroblast growth factor-23 (FGF-23), known for its role in mineral metabolism disturbances, is associated with CKD progression and increased cardiovascular risk. Developing composite biomarker panels-combining markers such as SCr, Cystatin C, and the albumin-to-creatinine ratio-further enhances risk stratification for CKD progression and mortality [9]. Prognostic biomarkers are crucial for assessing CKD patients' mortality risk and cardiovascular events. Inflammatory markers like IL-6 and CRP have been linked to higher mortality risk due to their role in systemic inflammation. In addition, cardiovascular risk markers such as Growth Differentiation Factor-15 (GDF-15) predict cardiovascular events in CKD patients. Another key biomarker, soluble ST2, is associated with heart failure outcomes and can predict mortality in individuals with CKD [43]. Predicting treatment response is another vital aspect of CKD management. Biomarkers like KIM-1 and NGAL can help assess the effectiveness of interventions such as RAAS inhibitors or dietary modifications by tracking changes in kidney injury status over time. Emerging research suggests that metabolomic profiles may offer insights into individual treatment responses, paving the way for more personalized CKD management. Integrating multi-omic approaches, which combine genomic, proteomic, and metabolomic data, promises to create comprehensive biomarker panels that predict treatment responses more accurately than traditional markers alone. This holistic approach could greatly enhance precision in CKD management for older adults [44]. A summary of biomarkers for predicting CKD progression and outcomes in older adults is shown in Table 3.

**Table 3:** Biomarkers for predicting chronic kidney disease (CKD) progression and outcomes in older adults

Biomarker	Description	Clinical Relevance	Limitations
Serum Creatinine and Estimated Glomerular Filtration Rate (GFR) Decline [22]	Indicators of renal function decline over time.	Widely used for assessing CKD progression.	Poor sensitivity for early changes, influenced by age and muscle mass.
Albuminuria/Proteinuria [45]	Increased excretion of albumin or proteins in urine.	An early marker of kidney damage and predictor of CKD progression.	It can be affected by transient factors like exercise and fever.
Serum Cystatin C [46]	A more stable marker of GFR compared to creatinine.	Predicts risk of CKD progression, cardiovascular events, and mortality.	Limited routine availability; cost considerations.
Plasma Fibroblast Growth Factor-23 (FGF-23) [36]	Regulates phosphate and vitamin D metabolism.	High levels are associated with CKD progression, cardiovascular events, and mortality.	Influenced by bone and mineral disorders not specific to CKD.
Serum Beta-2 Microglobulin (β2M) [47]	Low molecular weight protein filtered by glomerulus.	Predicts all-cause mortality and CKD progression in older adults.	Elevated in other conditions such as inflammation and malignancies.
N-terminal pro B-type Natriuretic Peptide (NT-proBNP) [48]	A marker of cardiac stress and volume overload.	Predicts cardiovascular outcomes and mortality in CKD patients.	Influenced by cardiac and non-cardiac conditions.
Serum Interleukin-6 (IL-6) [49]	A pro-inflammatory cytokine is elevated in CKD.	Associated with inflammation, CKD progression, and mortality risk.	Non-specific; elevated in various inflammatory conditions.
High-sensitivity C-reactive protein (hs-CRP) [50]	A marker of systemic inflammation.	Predicts cardiovascular events and all-cause mortality in CKD.	Low specificity; affected by acute infections and inflammatory states.
Klotho [51]	A protein that regulates phosphate metabolism and vascular health.	Low levels associated with CKD progression and cardiovascular events.	Limited clinical use; influenced by aging and other comorbidities.
Serum TNF Receptors (sTNFR1, sTNFR2) [40]	Soluble forms of TNF receptors reflect inflammatory activity.	Predicts CKD progression and mortality in older adults.	Elevated in systemic inflammation; not kidney-specific.
Uromodulin [52]	A protein produced by renal tubules.	Lower levels are associated with an increased risk of CKD progression.	Limited clinical use; influenced by genetic factors.

Emerging technologies in CKD biomarker discovery

Emerging technologies are significantly advancing the discovery of biomarkers for CKD by leveraging multi-omics approaches, artificial intelligence (AI), and machine learning (ML) to enhance clinical applications. The integration of multi-omics data-encompassing genomics, transcriptomics, proteomics, and metabolomics-is essential for a comprehensive understanding of CKD. These technologies enable high-throughput analysis of biological samples, allowing researchers to identify novel biomarkers that reflect the complex interactions within biological systems [53]. For example, advancements in genomics facilitated by AI algorithms, such as convolutional neural networks (CNNs), enhance the identification of genomic variants, improving diagnostic accuracy. In proteomics, AI tools like MaxQuant facilitate advanced protein analysis, while AlphaFold predicts protein structures, aiding drug discovery efforts. Similarly, in metabolomics, machine learning techniques analyze metabolic profiles to uncover disease-associated signatures, potentially leading to early diagnosis and personalized treatment strategies. The combination of these omics data types provides a holistic view of CKD progression and enables the identification of biomarkers that are sensitive and specific to various stages of the disease [54]. AI and ML are transforming biomarker discovery by analyzing the vast datasets generated from multi-omics studies. These technologies excel at identifying patterns and correlations that traditional statistical methods may overlook. One key area of predictive modeling allows AI to integrate diverse datasets to create models that identify potential biomarkers associated with CKD progression. This includes supervised learning methods that train on labeled datasets to predict outcomes based on input features from omics data [55]. Moreover, the emergence of explainable AI (XAI) provides insights into the decision-making processes of AI models, enhancing trust in their predictions and facilitating further validation studies. The application of AI in CKD biomarker research not only improves accuracy but also accelerates the discovery process by enabling researchers to handle large-scale data efficiently [56]. The ultimate goal of these advancements is to integrate identified biomarkers into clinical practice, paving the way for personalized medicine in CKD management. This involves developing tailored treatment plans based on individual biomarker profiles, allowing clinicians to target specific pathways involved in CKD progression. Additionally, digital biomarkers and AI-driven analytics can facilitate continuous monitoring of patient health metrics, enabling timely interventions based on real-time data [57]. The combination of multiple biomarkers through AI algorithms enhances diagnostic precision, enabling earlier detection and intervention strategies for CKD patients. In conclusion, the convergence of multi-omics technologies with AI and ML is revolutionizing CKD biomarker discovery. This synergy not only enhances our understanding of disease mechanisms but also holds promise for developing personalized treatment approaches that could significantly improve patient outcomes [53]. A summary of emerging technologies in CKD biomarker discovery is presented in Table 4.

**Table 4:** Emerging technologies in chronic kidney disease (CKD) biomarker discovery

Technology	Description	Applications in CKD	Advantages	Limitations
Proteomics [58]	Large-scale study of proteins, including their structures and functions.	Identification of novel protein biomarkers related to CKD progression and kidney damage.	High-throughput, comprehensive protein profiling.	High cost, complex data analysis, and variability in sample quality.
Metabolomics [59]	Study of small molecules (metabolites) in biological samples.	Identification of metabolic alterations and discovery of CKD-specific biomarkers.	Provides insights into metabolic pathways involved in CKD.	Sensitivity to pre-analytical variations; complex data interpretation.
Genomics [60]	Study of the complete set of DNA, including genes and their functions.	Identification of genetic variants associated with CKD susceptibility and progression.	Enables personalized medicine approaches.	Ethical concerns, high cost, and need for large cohorts.
Transcriptomics [61]	Study of RNA transcripts produced by the genome under specific conditions.	Understanding gene expression changes in CKD and identification of RNA biomarkers.	Provides insights into gene regulation and disease mechanisms.	High variability requires high-quality samples.
Single-cell RNA Sequencing (scRNA-seq) [62]	Analysis of gene expression at the single-cell level.	Identification of cell-specific biomarkers and understanding cell heterogeneity in CKD.	High resolution reveals cell-specific changes.	High cost, complex data analysis, and limited clinical application.
Exosome Analysis [63]	Study of small extracellular vesicles containing proteins, lipids, and RNA.	Identification of exosome-derived biomarkers for early detection and prognosis of CKD.	Non-invasive, potential for early diagnosis.	Requires standardization, complex isolation, and analysis procedures.
Microbiome Analysis [64]	Study of the microbial communities in the body, particularly in the gut.	Exploring gut-kidney axis and identifying microbiome-related biomarkers for CKD.	Provides insights into host-microbe interactions in CKD.	Complex data interpretation, influenced by diet and environment.
Artificial Intelligence (AI) and Machine Learning (ML) [65]	Use of AI/ML algorithms to analyze large datasets and identify patterns.	Predictive modeling for CKD progression and discovery of novel biomarker patterns.	Ability to handle large and complex datasets; improves prediction accuracy.	It requires large datasets, has a risk of overfitting, and needs validation.
CRISPR/Cas9 Genome Editing [66]	Gene-editing technology for modifying specific DNA sequences.	Functional validation of genetic biomarkers and identification of novel therapeutic targets in CKD.	High precision and specificity in gene editing.	Ethical concerns, off-target effects, and technical challenges.
Liquid Biopsy [67]	Analyse circulating biomarkers in body fluids (e.g., blood, urine).	Non-invasive detection of CKD biomarkers and disease monitoring.	Minimally invasive, real-time monitoring of disease progression.	Limited sensitivity for early-stage disease requires validation.

Challenges and limitations of biomarkers in older adults with CKD

Using biomarkers to assess CKD among older adults presents several challenges and limitations. These issues arise from biological variability, differences in performance across disease stages, economic factors, and the need for more extensive longitudinal studies [68]. One primary challenge is the variability in biomarker levels due to age-related factors. Biomarkers can exhibit significant fluctuations resulting from physiological changes associated with aging. Research indicates that many standard clinical biomarkers may not accurately reflect health status in older adults, as their predictive power often diminishes with age [69]. For instance, age-related changes can alter biomarker levels in ways that do not necessarily correlate with disease progression or severity, complicating the interpretation of results. Furthermore, the aging process is non-linear and can vary significantly between individuals, making it difficult to establish standardized reference ranges for older populations [69]. Another significant limitation is the differences in biomarker performance across various stages of CKD. The effectiveness of biomarkers can vary considerably depending on the disease stage. Some biomarkers may indicate more early-stage kidney dysfunction, while others may be better suited for advanced stages. This variability necessitates careful selection of biomarkers tailored to the specific CKD stage being evaluated. Additionally, many emerging biomarkers lack sufficient validation across diverse populations and CKD stages, limiting their clinical utility and raising concerns about their practice reliability [42]. Cost and accessibility also pose considerable barriers to implementing novel biomarkers. While advanced biomarkers show promise for improving CKD management, many require sophisticated testing methods that may not be readily available in all clinical settings. The economic implications of incorporating these tests can be prohibitive for both healthcare systems and patients. Therefore, assessing the clinical utility of these biomarkers is crucial to ensure they provide meaningful benefits relative to their costs, especially in resource-limited settings [42]. Lastly, there is a pressing need for longitudinal studies that specifically focus on older adults with CKD. Such studies are essential to understand how biomarker levels change over time within this demographic and how these changes correlate with clinical outcomes. Current research often relies on cross-sectional data, which may not capture the dynamic nature of biomarker changes associated with aging and disease progression. Longitudinal studies could provide valuable insights into the predictive power of various biomarkers and help establish robust guidelines for their use in clinical practice [70]. A summary of the challenges and limitations of biomarkers in older adults with CKD is presented in Table 5.

**Table 5:** Challenges and limitations of biomarkers in older adults with chronic kidney disease (CKD)

Challenge/ Limitation	Description	Impact on Biomarker Utility	Potential Solutions
Age-related Variability [70]	Biomarker levels can vary significantly with age due to physiological changes.	Reduced specificity and accuracy in predicting CKD progression.	Age-adjusted reference ranges; combining multiple biomarkers.
Comorbid Conditions [71]	High prevalence of comorbidities (e.g., diabetes, cardiovascular disease).	Confounding effects on biomarker levels make it difficult to isolate CKD-specific signals.	Use of multimodal diagnostic approaches to distinguish between conditions.
Polypharmacy [72]	Multiple medications can alter biomarker levels or interfere with their measurement.	Drug-induced alterations may obscure true kidney function or damage.	Comprehensive medication review and adjustment for pharmacological effects.
Frailty and Sarcopenia [73]	Reduced muscle mass and frailty can affect biomarkers like creatinine.	Inaccurate estimation of kidney function leads to misclassification of CKD stage.	Use alternative biomarkers like cystatin C; incorporate frailty indices.
Inflammation and Immune Dysregulation [74]	Chronic inflammation and altered immune response are common in older adults.	Elevation of inflammatory biomarkers unrelated to CKD (e.g., C-reactive protein (CRP), Interleukin-6 (IL-6)).	Identification of CKD-specific inflammatory markers; adjustment for inflammation.
Nutritional Status [75]	Malnutrition or altered dietary intake can affect biomarker levels.	Variability in protein biomarkers like albumin and urea.	Assessment and correction of nutritional status before biomarker interpretation.
Cognitive Impairment [76]	Impaired cognitive function can lead to poor adherence to diagnostic procedures.	Inconsistent or unreliable sample collection (e.g., 24-hour urine collection).	Simplification of diagnostic procedures; use of non-invasive and easy-to-collect samples.
Biological Heterogeneity [77]	Genetic, epigenetic, and environmental differences influence biomarker levels.	Difficulty in establishing universal cut-offs and interpretation guidelines.	Personalized medicine approaches; stratification based on genetic and environmental factors.
Sampling and Measurement Issues [78]	Variability in sample collection, processing, and storage.	Potential pre-analytical errors and inconsistency in biomarker measurement.	Standardization of protocols; use of automated and reliable assays.
Limited Validation in Older Populations [79]	Most biomarkers are validated in younger or general populations, not specific to older adults.	Lack of age-specific data leads to reduced clinical applicability.	Conducting age-specific studies and clinical trials for biomarker validation.
Cost and Accessibility [80]	High cost and limited availability of advanced biomarkers in routine clinical practice.	Restriction in widespread use and real-world applicability.	Development of cost-effective assays; integration into routine testing panels.

Future directions

The field of CKD is rapidly evolving, particularly with advancements in biomarker research. One of the most promising areas is the development of non-invasive biomarker detection methods. Recent innovations in technologies such as liquid biopsies and infrared spectroscopy pave the way for detecting biomarkers in bodily fluids like urine and blood without invasive procedures. These non-invasive methods provide reliable, real-time insights into kidney health, potentially allowing for earlier intervention and better management of CKD. By minimizing patient discomfort and risk, these advancements could significantly enhance clinical approaches to monitoring kidney function [42]. Another crucial direction in CKD biomarker research is the exploration of combination biomarker panels. There is a growing recognition that relying on a single biomarker may not adequately capture the complexity of CKD. Researchers are investigating the use of multiple biomarkers that can be integrated to enhance diagnostic accuracy and prognostic capabilities. This approach could develop composite scores that reflect kidney function and damage more effectively, thereby improving patient risk stratification. Such panels could provide a more comprehensive understanding of an individual's kidney health, ultimately guiding more informed clinical decisions [9]. Personalized medicine is also becoming increasingly relevant in the management of CKD. Healthcare providers can tailor interventions based on individual patient profiles by leveraging genomic, proteomic, and metabolomic data. This personalized approach aims to optimize treatment efficacy while minimizing adverse effects by targeting specific biological pathways involved in kidney disease progression. As our understanding of genetic predispositions and individual responses to treatment grows, personalized biomarker-based interventions could revolutionize how CKD is managed, leading to better patient outcomes [57]. Finally, ongoing clinical trials are essential for validating novel biomarkers and assessing their clinical utility, specifically in older adults with CKD. These studies focus on evaluating the effectiveness of new biomarkers in predicting disease progression and treatment responses. By integrating advanced statistical methods and machine learning techniques, researchers can enhance the evaluation of these biomarkers, paving the way for their incorporation into routine clinical practice. The insights gained from these trials will be crucial for developing evidence-based guidelines that improve early diagnosis, treatment strategies, and overall patient outcomes in chronic kidney disease management [68].

## Conclusions

CKD in older adults presents a complex challenge due to the interplay of age-related changes, comorbidities, and the gradual progression of the disease. Early detection and precise monitoring are critical to mitigating the adverse outcomes associated with CKD, such as cardiovascular complications and progression to end-stage renal disease. Traditional biomarkers, while useful, have limitations, particularly in aging populations, necessitating the development of advanced biomarkers that can more accurately reflect kidney function and damage. These advanced biomarkers, which encompass inflammation, fibrosis, oxidative stress, and tubular injury markers, hold significant promise for improving early diagnosis, predicting disease progression, and guiding personalized treatment approaches in older adults. Despite their potential, challenges remain, including variability in biomarker levels and the need for further research to establish their clinical utility in this population. As advancements in technology, such as multi-omics and artificial intelligence, continue to drive biomarker discovery, integrating these tools into clinical practice could revolutionize CKD management in older adults, offering new pathways for improving patient outcomes and quality of life.
